# Identification of specific neutralizing antibodies for highly pathogenic avian influenza H5 2.3.4.4b clades to facilitate vaccine design and therapeutics

**DOI:** 10.1080/22221751.2024.2302106

**Published:** 2024-01-03

**Authors:** Bao Tuan Duong, Seon Ju Yeo, Hyun Park

**Affiliations:** a Zoonosis Research Center, Department of Infection Biology, School of Medicine, Wonkwang University, Iksan, Korea; b Department of Tropical Medicine and Parasitology, Department of Biomedical Sciences, College of Medicine, Seoul National University, Seoul, Republic of Korea; c Department of Tropical Medicine and Parasitology, Medical Research Center, Institute of Endemic Diseases, Seoul National University, Seoul, Republic of Korea

**Keywords:** H5n6, 2.3.4.4b Subclade, highly pathogenic avian influenza, epitope mapping, protein-protein docking, combination therapy

## Abstract

The highly pathogenic avian influenza H5 2.3.4.4 and 2.3.2.1c subclades have distinct antigenic properties and are responsible for the majority of human infections. Therefore, it is essential to understand the processes by which antibodies inhibit these subclade viruses to develop effective therapies and vaccines to prevent their escape from neutralizing antibodies. Herein, we report the epitopes of two specific monoclonal antibodies (mAbs) targeting haemagglutinin (HA) of the H5 2.3.4.4b subclade and their neutralizing abilities. The results indicated that the two mAbs provided specific protection against the H5 2.3.4.4b clade viral challenge in MDCK cells and mouse models. Through epitope identification and docking studies, we showed that these novel sites (which are located near the 130-loop (S136, T143) and 190-helix (N199, N205) of HA receptor-binding sites that contribute to the binding affinity of neutralizing mAbs and six residues of the complementarity-determining regions) can be targeted to generate antibodies with enhanced cross-neutralization. This can also help in understanding escape mutations that differ among the H5 2.3.4.4b, h, and 2.3.2.1c subclades. These results provide specific information to facilitate future vaccine design and therapeutics for both subclade viruses, which are dominant and pose a serious threat to humans.

## Introduction

Highly pathogenic avian influenza virus (HPAIVs) (H5N1) A/goose/Guangdong/1/1996 lineage viruses cause high morbidity and mortality (> 50% mortality) worldwide [1]. Since its first detection during an outbreak in Guangdong Province, China, in 1996, HPAI H5N1 has evolved into ten clades (0–9) with multiple subclades based on the diverse occurrence of haemagglutinin (HA) genes in the H5N1–9 subtypes [2,3]. To date, the HPAIV H5Nx 2.3.4.4 (b, h) and 2.3.2.1c subclades have been responsible for the majority of potential infection risks, accounting for 97% and 0.2% worldwide, respectively [4]. In addition, from 2020 to 2022, there have been six reported cases of human infections with influenza H5 2.3.4.4b clades, including one death [5].

Vaccination is a valuable preventative tool for the current influenza viral infection [6,7]. However, owing to the antigenic drift and shift in HA genes, the vaccine is less effective at neutralization and against newer circulating HPAIV H5 clades [8–10]. Therefore, the therapeutic use of monoclonal antibodies (mAbs) is a good alternative to vaccines for influenza during the early infectious stages [11,12].

The virus enters the host cell through the receptor-binding sites (RBS) linked to the globular head region of HA via endocytosis, resulting in a pH-dependent entry through endosomal fusion. Viral RNA is then released into the cytoplasm, reaching the nucleus, where it is transcribed for viral replication [13,14]. Many mAbs reportedly induce virus neutralization, mainly targeting the RBS of the HA protein of the influenza virus, including the HPAV H5Nx clades [12,15,16]. However, most of them targeted the ancestral virus lineages but not the most commonly circulating HPAV H5Nx clades 2.3.4.4b and 2.3.2.1c [15,17,18]. Recently, Schuele et al. reported murine monoclonal antibodies that both protect and neutralize the HA of influenza H5 clades 2.3.2.1 and 2.3.4.4; however, the epitope and antibody sequences were not identified in detail [19].

In our previous study, two novel (#11.4 and #23.3) specific mAbs targeting the HA of the H5N6 2.3.4.4b subclade viruses were developed and applied to diagnostic systems [20]. Here, we report the neutralization abilities of both mAbs against the H5 2.3.4.4b clade viral challenge in mouse models and Madin-Darby Canine Kidney cell line (MDCKs). Moreover, we found that both mAbs targeted the RBS of the HA of the H5 2.3.4.4b subclade and eight escape mutants on the 130-loop and 190-helix RBS of HA that contribute to the distinct antigenic sites among HPAIV H5 subclades 2.3.4.4b, h, and 2.3.2.1c. Furthermore, we used docking analysis to determine whether parts of the complementarity-determining regions (CDRs) should be engineered to produce antibodies with improved cross-neutralization.

## Materials and methods

### Cells and viruses

MDCK cells were obtained from the American Type Culture Collection (Manassas, VA, USA). 293 T cells were generously provided by Dr. Chris MOK from The University of Hong Kong. Recombinant H5 viruses containing HA from A/Anas/KR/2017/2.3.4.4b, RG/A/Swan/MG/2020/2.3.4.4 h, and RG/A/VN/2014/2.3.2.1c, referred to as wild-type viruses (Table S1), and internal gene segments from A/Puerto Rico/8/34 (H1N1) (PR8) were generated as previously described [20,21]. The recommended H5 mutants of A/Anas/KR/2017/2.3.4.4b and RG/A/Swan/MG/2020/2.3.4.4 h were generated via site-directed mutagenesis of the plasmids using the primers described in Table S2. Plasmids were transfected into a mixture of MDCK and 293 T cells to rescue viruses. Rescued viruses were amplified in 10-day-old embryonated chicken eggs, and virus stocks were stored at −80 °C.

### In vitro microneutralisation assay

*In vitro* microneutralisation assays were performed as previously described [22]. Briefly, 60 µL of serial two-fold dilutions of mAbs (160 μg/mL or lower) or positive control (chicken antiserum) were combined with 60 µL of 100 × 50% tissue culture infectious dose per 50 μL (100× TCID50/50 μL) of viruses and incubated for 1 h at 37 °C. The mixture was transferred to a 96-well plate containing 2 × 10^4^ MDCK cells per well. After 1 h of adsorption, the viral inoculum was removed, and the plates were washed with phosphate-buffered saline (PBS). The cells were cultivated in Dulbecco's Modified Eagle Medium containing corresponding mAbs and supplemented with 2 µg/mL trypsin. The cells were incubated for 48 h at 37 °C and 5% CO_2_. Subsequently, the supernatant was collected for haemagglutination assay, which was used to determine neutralization. A volume of 50 µL of 0.75% chicken red blood cells was mixed with an equal volume of cell culture supernatant. The resulting mixture was incubated at room temperature for 30 min. Minimal serial dilutions of the positive control that resulted in haemagglutination were used to calculate neutralization titres. For the inhibitory concentration 50% (IC_50_), agglutination wells were marked, and IC_50_ values were calculated using GraphPad Prism Software Version 9.5.0. The experiment was performed in triplicate.

### Animal study

The prophylactic and therapeutic efficacies of the mAbs were evaluated using a mouse model following a previously described methodology with modifications [12]. Briefly, in the prophylactic study, a group of 5 female BALB/c mice (8 weeks old) received 10, 5, 2.5, or 1 mg/kg of mAbs or 10 mg/kg of control IgG (200 μL) one day before being intranasally challenged with 5 times the 50% mouse lethal dose of A/Anas/KR/2017/2.3.4.4b viruses. In the therapeutic investigation, 20, 10, 5, or 1 mg/kg of mAbs or 20 mg/kg of control IgG were intravenously administered into the tail vein 24 h after viral infection. Daily mortality and morbidity rates were recorded. Body weight was assessed for up to 12 d following infection. In the combination-treated group, equal amounts of each antibody were combined to make the same concentration as in the single-treated group. These experiments were performed in conformity with all good laboratory practice recommendations and were authorized by the Wonkwang University Committee on the Ethics of Animal Experiments (approval number: WKU23-52; WKU23-51)

### Indirect ELISA

An indirect ELISA was performed as previously described [20,23]. Briefly, wild-type and mutated viruses were diluted to 1280 HAU/mL in coating buffer (bicarbonate/carbonate 100 mM, pH 9.6). Further, 100 µL of each virus was coated on a 96-well microtitre plate (Greiner GmbH, Pleidelsheim, Germany) and incubated overnight at 4 °C. The plates were rinsed with PBS containing 0.1% v/v Tween-20 (PBST) and blocked in PBST plus 5% non-fat milk for 2 h at 37 °C. For virus detection, 20 µg/mL of primary #11.4 or #23.3 mAbs and 0.2 μg/mL 100 μL/well of secondary IgG-HRP (Catalog Number: ab97046; Abcam, Cambridge, UK) was added and incubated for 1 h. After washing five times to remove nonspecific binding, 100 μL of WANTAI BioPharm tetramethylbenzidine substrate was added at room temperature for colour development for 10 min; subsequently, the reactions were stopped by adding 100 μL of 0.18 M sulphuric acid to each well. Optical density was measured at 450 nm using a microplate reader (SpectraMax® M Series Multi-Mode Microplate, San Jose, CA, USA).

### Monoclonal antibody sequencing and docking study

Monoclonal antibody variable sequences were obtained using SMART technology (https://www.takarabio.com/learning-centers/mrna-and-cdna-synthesis/cdna-synthesis/cloning-antibody-variable-regions) as previously described [24]. Mouse hybridoma clones were cultured and confirmed using an IsoStrip™ Mouse Monoclonal Antibody Isotyping Kit (11493027001; Roche Diagnostics, Rotkreuz, Switzerland). Total RNA was extracted from 10^6^ cells using a Macherey-Nagel™ NucleoProtect RNA kit (Macherey-Nagel™ 740400.50; Fisher Scientific, England, UK). Specific PCR primers and thermal conditions were selected based on a previous study. PCR products were cloned into a pCR®-Blunt II-TOPO® vector using the Zero Blunt™ TOPO™ PCR Cloning Kit and analysed via Sanger sequencing. The DNA sequence of each antibody-variable region was analysed using IgBLAST with default parameters, and the query organism was set to mouse (https://www.ncbi.nlm.nih.gov/igblast/) and SAbPred (https://opig.stats.ox.ac.uk/webapps/sabdab-sabpred/sabpred/abodybuilder2/). This analysis was conducted to determine the amino acid sequences and percentage identity between the antibody-variable regions and reference sequences provided by IgBLAST for both light and heavy chains. To get the 3D-structures of antibody-variable regions, the final amino acid sequence for each antibody was uploaded to the I-TASSER server (https://zhanggroup.org/I-TASSER/) (Table S3). Further, protein–protein docking was performed using the LZerD Web Server with epitope site constraint and adjusted parameters for residue-residue distance (0 to 3 Å) (https://lzerd.kiharalab.org/upload/). The PyMOL Molecular Graphics System was used to view residues that interacted with mAbs and antigens (https://pymol.org/2/).

## Results

### Binding activity and specific neutralizing of mAbs to H5 2.3.4.4b subclade virus in vitro

**Figure 1.**
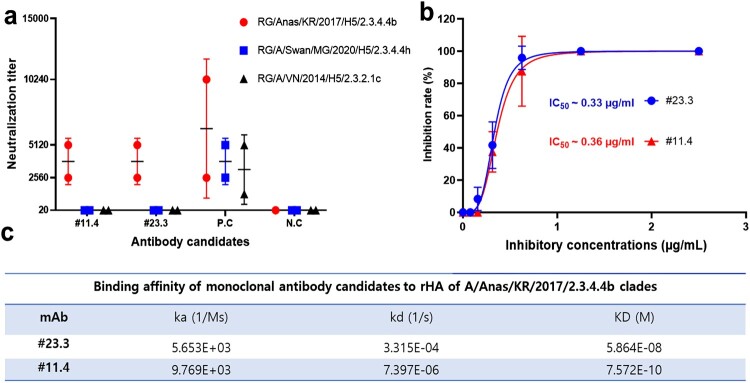
*In vitro* neutralization and binding activity of #23.3 and #11.4 mAbs. (a) Microneutralisation assay of #23.3 and #11.4 mAb candidates against the RG-H5 influenza virus, including the 2.3.4.4b, h, and 2.3.2.1c subclades. (b) Inhibitory concentration 50% (IC_50_) values of #23.3 and #11.4 mAb candidates against the RG-H5-HPAI-A/Anas/KR/2017/2.3.4.4b strain. Data represent the average values from at least two independent experiments (Figure S1 and S2) and are marked with different colours and symbols. IC_50_ values were calculated using GraphPad Prism Software Version 9.5.0. (c) Binding affinity of monoclonal antibody candidates to purified HA proteins of the A/Anas/KR/2017/2.3.4.4b subclades. The experiment was performed using Biacore T200 Control software version 3.2 (GE Healthcare, Danderyd, Sweden) and CM5 chip (GE healthcare, Cat#: BR-1005-30). KD, equilibrium dissociation rate constant; ka, association rate constant; kd, dissociation rate constant. Both results were presented as the mean ± SD

To evaluate and characterize the activity of #11.4 and #23.3 mAbs against influenza A viruses, we first performed microneutralisation assays of both mAbs against three subclades of influenza A virus H5 strains: 2.3.4.4b, 2.3.4.4 h, and 2.3.2.1c (Figure 1a). We found that #11.4 and #23.3 mAbs could neutralize RG/A/Anas/KR/2017/2.3.4.4b (H5) subclade viruses. Interestingly, both mAbs showed specific neutralizing activities only against the RG/A/Anas/KR/2017/2.3.4.4b (H5) but not against the RG/A/VN/2014/2.3.2.1c (H5) and RG/A/Swan/MG/2020/2.3.4.4 h (H5) subclades. In contrast, the control antibody (chicken serum against H5 2.3.4.4b, h, and 2.3.2.1c subclades) reacted with all influenza A (H5) viruses. We then calculated the IC_50_ value of #23.3 and #11.4 mAbs against the RG/A/Anas/KR/2017/2.3.4.4b (H5) subclade (Figure 1b). We found that #23.3 and #11.4 mAbs showed high reactivity with the RG/A/Anas/KR/2017/2.3.4.4b (H5) subclade, with IC_50_ values of 0.33 and 0.36 µg/mL, respectively. Further, we used surface plasmon resonance to measure the binding affinity of both #23.3 and #11.4 mAbs against purified HA proteins of the A/Anas/KR/2017/2.3.4.4b subclade. #11.4 mAb showed strong affinity for RG/A/Anas/KR/2017/2.3.4.4b (H5) HA, with a lower affinity constant (KD) of 0.7572 nM, whereas #23.3 mAb showed a ∼77-fold higher affinity constant (KD) than #11.4 mAb (58.64 nM) (Figure 1c). Taken together, these results indicated that both #23.3 and #11.4 mAbs presented specific neutralizing activities against the RG/A/Anas/KR/2017/2.3.4.4b (H5) subclade with comparable IC_50_ values, and that the binding affinity of #11.4 mAb was stronger than that of #23.3 mAb.

### In vivo prophylactic and therapeutic efficacy of mAbs in mouse model

**Figure 2.**
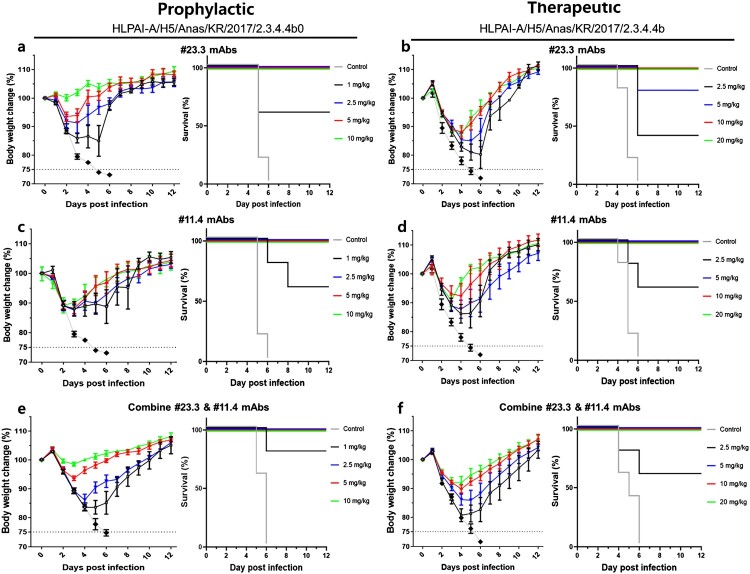
*In vivo* prophylactic and therapeutic efficacy of #23.3 and #11.4 mAbs in mouse model. (a, c) Prophylactic efficacy of #23.3 and #11.4 mAbs against lethal challenge of the RG-H5-HPAI-A/Anas/KR/2017/2.3.4.4b strain. Body weight (left) and survival (right) curves of BALB/c mice (n = 5 per group) treated with 10, 5, 2.5, or 1 mg/kg #23.3 or #11.4 mAb (control IgG of 10 mg/kg normal mouse) 24 h before lethal challenge are shown. (b, d) Therapeutic efficacy of #23.3 and #11.4 mAbs against lethal challenge of the RG-H5-HPAI-A/Anas/KR/2017/2.3.4.4b strain. (e, f) Prophylactic and therapeutic efficacy of combination #23.3 & #11.4 mAbs against lethal challenge of the RG-H5-HPAI-A/Anas/KR/2017/2.3.4.4b strain. BALB/c mice (n = 5 per group) treated with 20, 10, 5, 2.5, or 1 mg/kg #23.3 or #11.4 mAb (control IgG of 20 mg/kg normal mouse) 24 h after lethal challenge are shown. Data are presented as the means ± SEM.

We investigated the prophylactic and therapeutic efficacies of both mAbs against RG/A/Anas/KR/2017/2.3.4.4b (H5) viral infections in a mouse model. Groups of 8-week-old female BALB/c mice (n = 5) were intranasally administered five lethal doses (LD_50_) of RG/A/Anas/KR/2017/2.3.4.4b (H5) viral infection in a mouse model, and antibodies (dosage range of 1–20 mg/kg) were administered 24 h before and after viral infection for the prophylactic and therapeutic groups, respectively. The results showed that in the prophylactic groups (n = 5), doses of at least 2.5 mg/kg of both mAbs could completely protect mice from a lethal infection with RG/A/Anas/KR/2017/2.3.4.4b (H5). Further, these doses of #23.3 mAb had less impact on mouse body weight compared with the #11.4 mAb (Figure 2a, c). In addition, doses as low as 1 mg/kg provided 60% survival for both #23.3 and #11.4 mAbs. In the therapeutic groups (n = 5), 5 mg/kg and above of #11.4 mAb could fully protect the mice against the RG/A/Anas/KR/2017/2.3.4.4b (H5) virus, whereas a higher dose of 10 mg/kg and above of #23.3 mAb provided similar protection against a lethal infection with RG/A/Anas/KR/2017/2.3.4.4b (H5) virus. The lower dosage of 2.5 mg/kg of both #23.3 and #11.4 mAbs offered partial protection, with a survival rate of 40% and 60%, respectively (Figure 2b, d). The administration of #23.3 mAb to therapeutic groups exhibited considerable changes in body weight, which were less pronounced than those in the prophylactic groups. Therefore, *in vivo* prophylactic and therapeutic results indicated that both #23.4 and #11.4 mAbs could protect mice against the RG/A/Anas/KR/2017/2.3.4.4b (H5) virus, although this protection was less effective in the therapeutic groups than in the prophylactic groups. We next investigate whether the combination treatment with #23.3 and #11.4 has any coaction to protect mice against the RG/A/Anas/KR/2017/2.3.4.4b (H5) virus (Figure 2e, f). In the prophylactic groups, treatment of mice with combined #23.3 and #11.4 mAbs (lower dosage of 1 mg/kg) resulted in an 80% survival rate. It increased the survival rate by 20% in comparison to a single treatment at the same dosage (1 mg/kg) of #23.3 and #11.4 (60%). In the therapeutic groups, this survival rate was obtained at 60% and 100% at lower dosages of 2.5 and 5 mg/kg, respectively. It was similar to that obtained with #11.4 monotherapy at dosages of 2.5 mg/kg (60%) but was higher than that obtained with #23.3 monotherapy at lower dosages of 2.5 mg/kg (40%) and 5 mg/kg (80%). In conclusion, combination therapy with both mAbs has some advantages in protecting mice on prophylaxis. However, in therapeutic treatment, combination therapy did not show any effectiveness compared to single treatment with mAbs for improving mouse survival rates.

### Epitope selection for validation via reverse genetics of influenza virus

**Figure 3.**
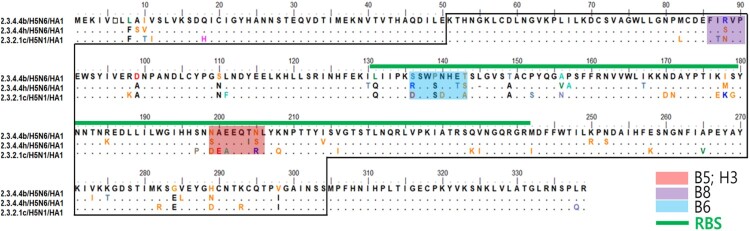
Alignment of amino acid sequences among HPAI H5 2.3.4.4b, h, and 2.3.2.1c clades. HA1 potential epitope sequences of mAb candidates are indicated by coloured rectangles; green lines indicate the HA receptor binding positions of HPAIV H5 viruses.

To determine the epitopes of #23.3 and #11.4 mAbs, we compared the amino acid sequences among three HPAIV H5 subclades, including RG/A/Anas/KR/2017/2.3.4.4b, RG/A/Swan/MG/2020/2.3.4.4 h, and RG/A/VN/2014/2.3.2.1c (Figure 3). Because both mAbs showed specific neutralizing activities against RG/A/Anas/KR/2017/2.3.4.4b, we selected three potential epitope regions (B5, H3, and B6) that belong to the RBS of HA, with high genetic variants among the three subclades. For comparison, we also selected a B8 region that did not belong to the RBS to generate mutated viruses via reverse genetics (RG). The site mutation of the epitopes was introduced via mutagenesis of plasmids following changes in amino acid sequences and primers (Table 1 and Table S2). The plasmids were then transfected into mammalian cells to rescue the RG-mutated viruses. RG viruses induced a cytopathic effect 4 days post-transfection (Figure S3). Finally, four mutated viruses were generated, and their sequences were confirmed using Sanger sequencing (http://www.cosmogenetech.com/main.jsp).

**Table 1.** List of reverse genetics viruses for validation of the epitopes of mAbs.

Wild-type viruses (Wt)			Mutated viruses (Mut)	
Labelling	Clades	Amino acid sequences	Labelling	Amino acid sequences*
Wt-B	PR8/H5/2.3.4.4b	199-NAEEQTN-205	Mut-B5	199-SAEEQIS-205
Wt-H	PR8/H5/2.3.4.4 h	199-SAEEQIS-205	Mut-H3	199-NAEEQTN-205
Wt-B	PR8/H5/2.3.4.4b	136-SSWPNHET-143	Mut-B6	136-DSWSDHEA-143
Wt-B	PR8/H5/2.3.4.4b	86-EFIRVP-90	Mut-B8	86-EFTNVP-90
Wt-C	PR8/H5/2.3.2.1c	86-EFTNVP-90		
		136-DSWSDHEA-143		

*Underlines indicate amino acids mutated from the wild-type viruses.

### Epitope validation via indirect ELISA

**Figure 4.**
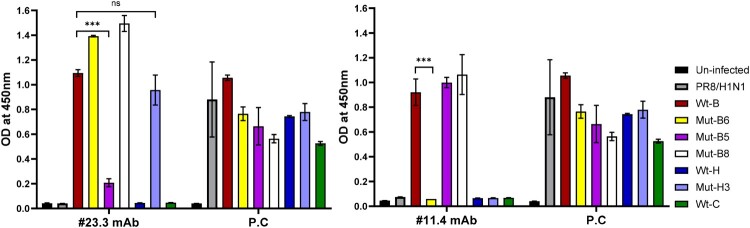
Epitope identification of specific neutralizing antibodies for highly pathogenic avian influenza H5 2.3.4.4b clades of the #23.3 and #11.4 mAbs via indirect ELISA. The means and standard deviation (SD) were calculated, and Student’s t-test was performed using GraphPad Prism Software Version 9.5.0 (La Jolla, CA, USA). Results are presented as the means ± SD. *** *p* < 0.001; ns, not significant; P.C., positive control anti-influenza A nucleoprotein.

**Figure 5.**
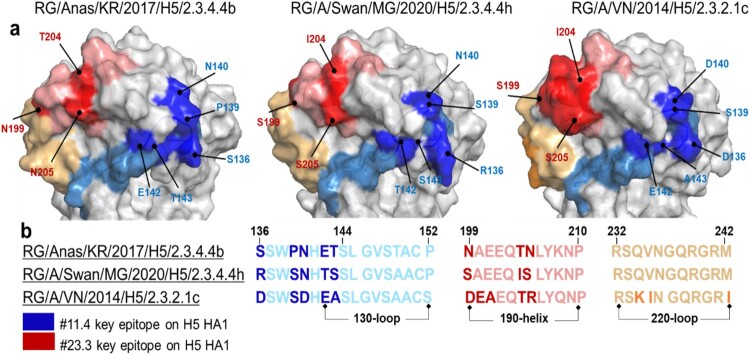
3D-modelling and alignment of epitopes of specific neutralizing antibodies on the HA head domain of three H5 virus clades. (a) Key residues of #11.4 and #23.3 mAbs on the HA of H5 viruses; the blue and red regions show all epitopes of #11.4 and #23.3 mAbs, respectively; key amino acid types are indicated with bold colours. (b) Alignment epitopes among three different H5 virus clades; numbering of the receptor binding sites with the 130-loop, 190-helix, and 220-loop are based on the HA of H3.

To validate the binding affinity of the antibody candidates to mutated viruses, we performed an indirect ELISA to compare wild-type (Wt) and mutated (Mut) viruses (Figure 4). The results showed that #23.3 and #11.4 mAbs strongly reacted to the Wt-B (2.3.4.4b subclade) virus but could not detect Wt-H (2.3.4.4 h subclade) and Wt-C (2.3.2.1c subclade). However, when we changed the amino acid number 199-NAEEQTN-205 of the Wt-B virus to 199-SAEEQIS-205 of the 2.3.4.4 h subclade (Mut-B5), the binding affinity of #23.3 mAb was significantly decreased (*** *p* < 0.001) for that virus. Interestingly, the binding affinity of #23.3 mAb was restored when the amino acid 199-SAEEQIS-205 of Wt-H (2.3.4.4 h subclade) was mutated to 199-NAEEQTN-205 of the 2.3.4.4b subclade (Mut-H3 virus). In contrast, #11.4 mAb failed to detect Mut-B6 (2.3.4.4b subclade) when the amino acid number 136-SSWPNHET-142 mutated to 136-DSWSDHEA-143 of the 2.3.2.1c subclade (*** *p* < 0.001), whereas the reactivity of #11.4 mAb remained for Wt-B, Mut-B5, and Mut-B8 viruses. In addition, 3D-modelling of the HA head domain showed that the surface epitopes of the three H5 virus clades were different, and the epitopes of #23.3 and #11.4 mAbs were far from each other (Figure 5a). In addition, alignment of epitope amino acid sequences showed that #23.3 mAb completely belonged to the 190-helix, whereas #11.4 mAb belonged to the 130-loop, partially in the RBS region (Figure 5b). Altogether, these results indicate that the amino acid positions N199S, T204I, and N205S seem to be key elements that contribute to the binding affinity of #23.3 mAb, and that S136D, P139S, N140D, and T143A contribute to the interaction between #11.4 mAb and the HA antigen target.

### Antibody sequencing and docking study

**Figure 6.**
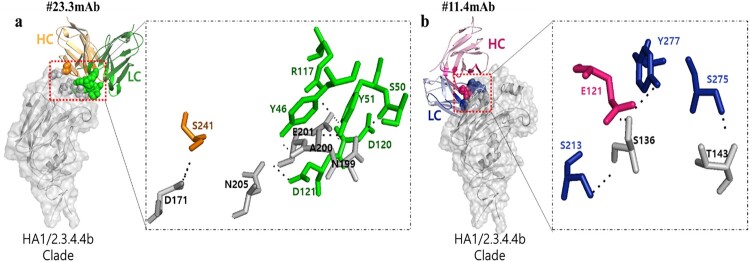
Prediction of hydrogen bonding interactions between antibody and antigen of the H5N6/HA1/A/Anas/KR/2017/2.3.4.4b clades using docking LZerD Web Server (https://lzerd.kiharalab.org/upload/). (a) Hydrogen bonding interactions of #23.3 mAb and HA1/2.3.4.4b clade; the heavy chain (HC) and light chain (LC) of #23.3 mAb are shown in bright orange and green colours, respectively. (b) Hydrogen bonding interactions of #11.4 mAb and HA1/2.3.4.4b clade; HC and LC of #11.4 mAb are shown in bright pink and blue colours, respectively. The probable hydrogen bonds are shown as black dashed lines. The binding sites of CDRs are highlighted in deep colours at both HC and LC of the mAb, and antigens sites are shown in bright grey colour. The mAb sequences for 3D-modelling are shown in Table S3.

DNA sequences corresponding to the variable region of the immunoglobulin light chain (VL) and heavy chain (VH) of samples #23.3 and #11.4 mAbs were obtained and compared with the nearest germline sequence using the IgBLAST database. These findings indicated that the sequence labelled #23.3 mAb corresponded to the specific germline IGKV4-74*01 and IGHV1S81*02 V genes for VL and VH, respectively. The nucleotide identity of the VL chain was 99.3% (290/292 nucleotides), whereas that of the VH chain was 98% (288/294 nucleotides). In addition, the alignment of #11.4 mAb corresponded to a specific IGKV9-120*02 for VL and IGHV1-63*02 germline V gene for VH, with a nucleotide identity of 99.6% (283/284 nucleotides) and 94.9% (279/294 nucleotides), respectively. We then performed protein–protein docking to predict the interaction between the mAbs and antigen target (H5N6/HA1/A/Anas/KR/2017/2.3.4.4b). The results showed that #23.3 mAb interacted widely with the antigen target, creating eight hydrogen bonds located in the RBS of the antigen, including seven contacts of HC-CRD1:CDR3 of the mAb with the 190-helix (N199, A200, E201, and N205) of the antigen target (Figure 6a and Table 2). In addition, only one hydrogen bond of LC/CDR2-S241 was in contact with the antigen residue D171. For #11.4 mAb, HC/CDR3, LC/CDR1, and LC/CDR3 formed four hydrogen bond contacts with two residues, S136 and T143, on the RBS of the antigen target (Figure 6b and Table 2). Interestingly, the S136 residue near the 130-loop was the main interaction, accounting for 75% (3/4) of the total hydrogen bonds. Generally, the hydrogen bonds of both mAbs with antigen targets were strong within 2.7 angstroms (**Å**) between the donor–acceptor atoms and binding sites on the RBS of the antigen target [25].

**Table 2.** Hydrogen bond network of single-chain variable fragment (scFv) and antigen of the H5N6/HA1/A/Anas/KR/2017/2.3.4.4b clades.

Monoclonal antibodies	scFv residue (atom)	Antigen (atom)	Distance (Å)
**#23.3**	LC/CDR2-S241	D171	2.5
	HC/CDR1-S50	N199	2.0
	HC/CRD1-Y46; CDR1-Y51	A200	0.9; 1.2
	HC/CDR1-Y51; CDR3-D120; CDR3-R117	E201	2.4; 2.5; 1.8
	HC/CDR3-D121	N205	2.3
**#11.4**	HC/CDR3-E121; LC/CDR1-S213; CDR3-Y277	S136	1.6; 2.5; 2.7
	LC/CDR3-S275	T143	1.8

LC, light chain; HC, heavy chain; CDR, complementarity-determining regions; Å, angstroms.

## Discussion

The spread of AIVs among wild birds has potential consequences for human health and the global poultry industry [26–30]. HPAIV H5 2.3.4.4 and 2.3.2.1c subclade infections in animals have jumped the species barrier and spread to humans [31–33]. To effectively battle HPAIV H5, universal vaccine candidates or broadly neutralizing antibodies targeting conserved epitopes are necessary [6,12,34]. However, the antigenic evolution of influenza viruses and HPAIV H5 subclades is a substantial barrier to the research and development of antiviral medicines and universal vaccines with high efficacies and long shelf lives [35]. Consequently, a therapeutic window using specific neutralizing antibodies would be useful for the current emergence and circulation of HPAIV H5 strains.

In this study, we analysed the biofunctionality and structure of #11.4 and #23.3 mAbs that specifically neutralized and targeted HA in H5 2.3.4.4b subclade viruses. We also analysed the key amino acids that contributed to the binding between mAbs and protein targets. In particular, both mAbs displayed significant neutralizing efficacy against strains of HPAI H5N1 2.3.4.4b subclade viruses in both *in vitro* and *in vivo* testing, and the binding sites of the mAbs may be located on the 130-loop and 190-helix RBS of the HA protein of the H5 2.3.4.4b subclades.

In a previous study on a cross-neutralizing antibody that targeted the HA of H1N1 and H5N1, the results revealed that IC_50_ values of 5.75 μg/mL and a dosage of 10 mg/kg or above of mAbs could completely protect mice from lethal infection of viruses in both prophylactic and therapeutic groups [12]. Schuele et al. found that mAbs completely neutralized the HPAI H5 2.3.4.4 and 2.3.2.1 subclades at concentrations between 0.3 and 20 μg/mL [19]. In our study, we obtained IC_50_ values of 0.33 and 0.36 µg/mL for #23.3 and #11.4 mAbs, respectively, and also found that doses of 10 mg/kg or higher of mAbs protected the mice from deadly viral infection. Therefore, our antibody is superior or comparable to previous reports on protection against and neutralization of viruses in mice.

The identification of mAb epitopes can be accomplished using various approaches, including crystal structure analysis, escape mutation, random mutagenesis library, and site-direct mutagenesis [36]. In this study, mAb epitopes were validated using site-directed mutagenesis and RG of influenza virus via indirect ELISA. The RBS structure is characterized by a cluster of conserved amino acids which are bordered by the 190-helix, 130-loop, and 220-loop [13]. Therefore, both mAbs were expected to directly block viral binding. However, additional experiments should be conducted to determine the binding mechanism of mAbs to the HA of H5 viruses.

Multiple studies have demonstrated that mAb epitopes that specifically target RBS have the potential to directly inhibit viral binding [11,12,16,18,19]. Five positions (S124, P127, N128, E130, and T131) that contribute to the binding affinity of #11.4 mAb overlap with the 130-loop RBS of the HA of the H5 2.3.4.4b subclade, such as C12H5, FLD21.140, 65C6, 3C11, and AVFlulgG01 mAbs, which reportedly neutralize ancestor virus lineages that include HPAIV H5; 2, 8, 7.1, and 1 clades [12,15,36]. Unlike #11.4 mAb, the binding affinity of #23.3 mAb is influenced by three specific positions (N190, T195, and N196) within the 190-helix. These positions exhibit significant spatial separation from the binding sites of #11.4 mAb, thereby precluding any epitope competition between the two mAbs. Furthermore, mutations occurring at these sites within the 190-helix did not alter the binding affinity of #11.4 mAb. Thus, the combined administration of our mAbs showed better efficacy than single treatment of mAbs against viral infections in prophylactic studies. This result implies that two mAbs recognize spatially distinct HA antigen domains, demonstrating modest complementarity in terms of their functionality to block viral entry. Therefore, the mAbs that recognize different antigen targets should be further investigated in order to determine the most effective and robust approach for preventing viral infection.

Docking studies involve the use of computational tools to predict binding sites within the complex structure of an antibody and antigen target or other molecules, thereby facilitating the identification of potential interactions [37–40]. In this study, the docking results suggested 12 potential interactions between CDRs and seven binding residues on the antigen target. Among these, HC/CDR1-S50 and HC/CDR3-D121 of #23.3 mAb, and HC/CDR3-E121, LC/CDR1-S213, CDR3-Y277, and LC/CDR3-S275 of #11.4 mAb may contribute to the binding specificity of mAbs because of their contact with non-conserved amino acids in 2.3.4.4b, h, and 2.3.2.1c, including N199, N205, S136, and T143. Therefore, the abovementioned sites on CDRs may serve as potential therapeutic targets to facilitate the development of modified antibodies with cross-neutralizing capabilities.

In addition to the significance of this research, there are some limitations that should be considered in this study. At first, this study only looked at a small number of specific neutralizing antibodies and didn't test to see if these sites were enough to make antibodies that could neutralize HPAIV H5. Therefore, the immunogenicity of escape mutations or incorporation of other parts should be validated before designing vaccines against H5Nx 2.3.4.4 and 2.3.2.1c subclades. The second restriction relates to the six amino acid residues of the CDRs, as anticipated by docking studies. Does this have a positive impact on the generation or modification of antibodies? To answer this question, one recombinant antibody (#23.3) was successfully produced and verified utilizing the E. coli expression method (data not shown in this study). After that, changes will be made to the CDRs to improve the antibody's affinity or the mAbs’ ability to cross-neutralize.

In conclusion, we identified a major mutation site in the RBS of H5 that contributes to viral escape from neutralizing antibodies among the different subclades 2.3.4.4b, h, and 2.3.2.1c. We found eight key points (S136, P139, N140, E142, T143, N199, T204, and N205) which may be useful during virus surveillance activities, for designing improved vaccines for the HPAIV H5Nx clades, particularly the 2.3.4.4b subclade, and for engineering antibodies that can efficiently destroy viruses and identify escape variants.

## Geological information

Asia, Europe, America.

## Supplementary Material

Supplementary_Information

## References

[1] World Health Organization. Avian Influenza Weekly Update Number 901 updated 23 June 2023. https://cdn.who.int/media/docs/default-source/wpro—documents/emergency/surveillance/avian-influenza/ai_20230623.pdf?sfvrsn = 5f006f99_116.

[2] https://nextstrain.org/. Last accessed 07 June 2023.

[3] Xu X, Subbarao K, Cox NJ, et al. Genetic characterization of the pathogenic influenza A/Goose/Guangdong/1/96 (H5N1) virus: similarity of its hemagglutinin gene to those of H5N1 viruses from the 1997 outbreaks in Hong Kong. Virology. 1999;261(1):15–19. doi:10.1006/viro.1999.9820 10484749

[4] Clade progression of H5Nx subtype from Jun 2020 to April 2023 Source: based on GISAID 2023. Last accessed 20 June 2023. https://platform.epicov.org.

[5] WHO. Assessment of risk associated with recent influenza A(H5N1) clade 2.3.4.4b viruses 21 December 2022. https://cdn.who.int/media/docs/default-source/influenza/avian-and-other-zoonotic-influenza/h5-risk-assessment-dec-2022.pdf?sfvrsn = a496333a_1&download = true.

[6] Beans C. Researchers getting closer to a “universal” flu vaccine. Proc Natl Acad Sci U S A. 2022;119(5). doi:10.1073/pnas.2123477119 PMC8812533 35082157

[7] Feranmi F. Universal flu vaccine protects against influenza A and B. Lancet Microbe. 2022;3(12):e902. doi:10.1016/S2666-5247(22)00293-2 36252577

[8] Chapter 25 - Vaccine-Preventable Diseases, in Immunology for Pharmacy, D.K. Flaherty, Editor. 2012, Mosby: Saint Louis. 197–213.

[9] Gu W, Shi J, Cui P, et al. Novel H5N6 reassortants bearing the clade 2.3.4.4b HA gene of H5N8 virus have been detected in poultry and caused multiple human infections in China. Emerg Microbes Infect. 2022;11(1):1174–1185. doi:10.1080/22221751.2022.2063076 35380505 PMC9126593

[10] Duong BT, Than DD, Ankhanbaatar U, et al. Assessing potential pathogenicity of novel highly pathogenic avian influenza (H5N6) viruses isolated from Mongolian wild duck feces using a mouse model. Emerg Microbes Infect. 2022;11(1):1425–1434. doi:10.1080/22221751.2022.2069515 35451353 PMC9154755

[11] Chen Y, Wang F, Yin L, et al. Structural basis for a human broadly neutralizing influenza A hemagglutinin stem-specific antibody including H17/18 subtypes. Nat Commun. 2022;13(1):7603. doi:10.1038/s41467-022-35236-y 36494358 PMC9734383

[12] Li T, Chen J, Zheng Q, et al. Identification of a cross-neutralizing antibody that targets the receptor binding site of H1N1 and H5N1 influenza viruses. Nat Commun. 2022;13(1):5182. doi:10.1038/s41467-022-32926-5 36056024 PMC9439264

[13] de Graaf M, Fouchier RA. Role of receptor binding specificity in influenza A virus transmission and pathogenesis. EMBO J. 2014;33(8):823–841. doi:10.1002/embj.201387442 24668228 PMC4194109

[14] Shi Y, Wu Y, Zhang W, et al. Enabling the ‘host jump': structural determinants of receptor-binding specificity in influenza A viruses. Nat Rev Microbiol. 2014;12(12):822–831. doi:10.1038/nrmicro3362 25383601

[15] Khurana S, Suguitan AL Jr, Rivera Y, et al. Antigenic fingerprinting of H5N1 avian influenza using convalescent sera and monoclonal antibodies reveals potential vaccine and diagnostic targets. PLoS Med. 2009;6(4):e1000049. doi:10.1371/journal.pmed.1000049 19381279 PMC2661249

[16] Zuo T, Sun J, Wang G, et al. Comprehensive analysis of antibody recognition in convalescent humans from highly pathogenic avian influenza H5N1 infection. Nat Commun. 2015;6:8855. doi:10.1038/ncomms9855 26635249 PMC4686829

[17] Lim AP, Wong SK, Chan AH, et al. Epitope characterization of the protective monoclonal antibody VN04-2 shows broadly neutralizing activity against highly pathogenic H5N1. Virol J. 2008;5:80. doi:10.1186/1743-422X-5-80 18616831 PMC2481255

[18] Okuda M, Yamayoshi S, Uraki R, et al. Subclade 2.2.1-specific human monoclonal antibodies that recognize an epitope in antigenic site A of influenza A(H5) virus HA detected between 2015 and 2018. Viruses. 2019;11(4). doi:10.3390/v11040321 PMC6521261 30987023

[19] Schuele C, Schmeisser F, Orr M, et al. Neutralizing and protective murine monoclonal antibodies to the hemagglutinin of influenza H5 clades 2.3.2.1 and 2.3.4.4. Influenza Other Respir Viruses. 2023;17(5):e13152. doi:10.1111/irv.13152 37246149 PMC10209644

[20] Duong BT, Than DD, Ju BG, et al. Development of a rapid fluorescent diagnostic system for early detection of the highly pathogenic avian influenza H5 clade 2.3.4.4 viruses in chicken stool. Int J Mol Sci. 2022;23(11). doi:10.3390/ijms23116301 PMC9181406 35682982

[21] Durairaj K, Than DD, Nguyen ATV, et al. Cysteamine-gold coated carboxylated fluorescent nanoparticle mediated point-of-care dual-modality detection of the H5N1 pathogenic virus. Int J Mol Sci. 2022;23(14). doi:10.3390/ijms23147957 PMC9320457 35887315

[22] Cuevas F, Kawabata H, Krammer F, et al. An in vitro microneutralization assay for influenza virus serology. Curr Protoc. 2022;2(7):e465. doi:10.1002/cpz1.465 35848945 PMC9298957

[23] Yeo SJ, Bao DT, Seo GE, et al. Improvement of a rapid diagnostic application of monoclonal antibodies against avian influenza H7 subtype virus using Europium nanoparticles. Sci Rep. 2017;7(1):7933. doi:10.1038/s41598-017-08328-9 28801679 PMC5554140

[24] Meyer L, Lopez T, Espinosa R, et al. A simplified workflow for monoclonal antibody sequencing. PLoS One. 2019;14(6):e0218717. 31233538 10.1371/journal.pone.0218717 PMC6590890

[25] Jeffrey GA. An introduction to hydrogen bonding. Oxford University Press; 1997; http://biomodel.uah.es/en/water/hbonds.htm.

[26] Duong BT, Bal J, Sung HW, et al. Molecular analysis of the avian H7 influenza viruses circulating in South Korea during 2018-2019: evolutionary significance and associated zoonotic threats. Viruses. 2021;13(11. doi:10.3390/v13112260 PMC8623559 34835066

[27] Nguyen NM, Sung HW, Yun KJ, et al. Genetic characterization of a novel North American-origin avian influenza A (H6N5) virus isolated from bean goose of South Korea in 2018. Viruses. 2020;12(7. doi:10.3390/v12070774 PMC7411716 32709116

[28] Trinh TT, Duong BT, Nguyen ATV, et al. Emergence of novel reassortant H1N1 avian influenza viruses in Korean Wild Ducks in 2018 and 2019. Viruses. 2020;13(1). doi:10.3390/v13010030 PMC7823676 33375376

[29] Trinh TT, Tiwari I, Durairaj K, et al. Genetic characterization and pathogenesis of avian influenza virus H7N3 isolated from spot-billed ducks in South Korea, early 2019. Viruses. 2021;13(5. doi:10.3390/v13050856 PMC8151380 34067187

[30] Yeo SJ, Hoang VT, Duong TB, et al. Emergence of a novel reassortant H5N3 avian influenza virus in Korean Mallard Ducks in 2018. Intervirology. 2022;65(1):1–16. doi:10.1159/000517057 34438407 PMC8820440

[31] Wille M, Barr IG. Resurgence of avian influenza virus. Science. 2022;376(6592):459–460. doi:10.1126/science.abo1232 35471045

[32] Zhang J, Ye H, Liu Y, et al. Resurgence of H5N6 avian influenza virus in 2021 poses new threat to public health. Lancet Microbe. 2022;3(8):e558. doi:10.1016/S2666-5247(22)00148-3 35750068

[33] Adlhoch C, Fusaro A, Gonzales JL, et al. Avian influenza overview December 2022 - March 2023. EFSA J. 2023;21(3):e07917. 36949860 10.2903/j.efsa.2023.7917 PMC10025949

[34] Isakova-Sivak I, Rudenko L. A promising candidate for a universal influenza vaccine. Lancet Infect Dis. 2023 Dec;23(12):1327–1329. doi:10.1016/S1473-3099(23)00366-3 37517421

[35] Lewis NS, Banyard AC, Essen S, et al. Antigenic evolution of contemporary clade 2.3.4.4 HPAI H5 influenza A viruses and impact on vaccine use for mitigation and control. Vaccine. 2021;39(29):3794–3798. doi:10.1016/j.vaccine.2021.05.060 34074548

[36] Wang P, Zuo Y, Sun J, et al. Structural and functional definition of a vulnerable site on the hemagglutinin of highly pathogenic avian influenza A virus H5N1. J Biol Chem. 2019;294(12):4290–4303. doi:10.1074/jbc.RA118.007008 30737282 PMC6433081

[37] Bao DT, Kim DTH, Park H, et al. Rapid detection of avian influenza virus by fluorescent diagnostic assay using an epitope-derived peptide. Theranostics. 2017;7(7):1835–1846. doi:10.7150/thno.18857 28638471 PMC5479272

[38] Kim DTH, Bao DT, Park H, et al. Development of a novel peptide aptamer-based immunoassay to detect Zika virus in serum and urine. Theranostics. 2018;8(13):3629–3642. doi:10.7150/thno.25955 30026871 PMC6037026

[39] Liu DX, Tien TTT, Bao DT, et al. A novel peptide aptamer to detect plasmodium falciparum lactate dehydrogenase. J Biomed Nanotechnol. 2019;15(1):204–211. doi:10.1166/jbn.2019.2667 30480527

[40] Nguyen ATV, Duong BT, Park H, et al. Development of a peptide aptamer pair-linked rapid fluorescent diagnostic system for Zika virus detection. Biosens Bioelectron. 2022;197:113768. doi:10.1016/j.bios.2021.113768 34763153

