# Supplementary material for: Identification of specific neutralizing antibodies for highly pathogenic avian influenza H5 2.3.4.4b clades to facilitate vaccine design and therapeutics
Source: Emerg Microbes Infect. 2024 Jan 3;13(1):2302106. doi: 10.1080/22221751.2024.2302106 (PMC10810642; doi:10.1080/22221751.2024.2302106)
Supplement: Supplementary_Information [file TEMI_A_2302106_SM7356.docx]

# Supplementary Information

**Supplementary Tables**

Table S1: List of reverse genetics viruses in this study.

Table S2: List of primers for inducing virus mutations.

Table S3. Amino acid sequences for 3D-modelling of the monoclonal antibodies.

**Supplementary Figures**

Figure S1. Haemagglutination results of microneutralisation assay.

Figure S2. Haemagglutination results of inhibitory concentration 50% (IC_50_) values.

Figure S3. Rescue of the HPAIV H5 virus clades that contain haemagglutinin (HA) mutation sites.

Table S1: List of reverse genetics viruses in this study.

| **Name** | **Subtype/clades** | **Genbank ID of HA** |
| --- | --- | --- |
| A/Vietnam/14011801/2014 | H5N1/2.3.2.1c | EPI624914^*^ |
| A/Puerto Rico/8/1934(H1N1) | PR8H1N1/- | AB671289.1^#^ |
| A/Anas platyrhynchos/Korea/W612/2017 | H5N6/2.34.4b | MG891804.1^#^ |
| A/Whooper Swan/Khuvsgul/#4/2020 | H5N6/2.3.4.4h | OK091117.1^#^ |

^#^Accessed by NCBI database, <https://www.ncbi.nlm.nih.gov/Structure/cdd/wrpsb.cgi>; *Accessed by GISAID database, <https://platform.epicov.org/epi3/frontend#384e22>.

HA, haemagglutinin

Table S2: List of primers for inducing virus mutations.

| Labelling | Sites of changed nucleotides | Primer sequences (5' to 3') |
| --- | --- | --- |
| B5 | A596G_ANTISENSE | TGCTCTTCTGCACTGTTGGAATGATGAATCCCCCAC |
|  | A596G_SENSE | GTGGGGGATTCATCATTCCAACAGTGCAGAAGAGCA |
|  | C611T_A614G_ANTISENSE | TTGGGTTTTTATAGAGACTTATCTGCTCTTCTGCACTGTTGGA |
|  | C611T_A614G_SENSE | CATCATTCCAACAGTGCAGAAGAGCAGATAAGTCTCTATAA |
| B6 | A406G_G407A_C415A_C416G_A418G_A427-ANTISENSE | GCTGTGCTCACCCCTAATGATGCTTCATGATCGCTCCAAGAATCCTTGGGGATGATCAGAATCTTC |
|  | A406G_G407A_C415A_C416G_A418G_A427-SENSE | GAAGATTCTGATCATCCCCAAGGATTCTTGGAGCGATCATGAAGCATCATTAGGGGTGAGCACAGC |
| B8 | T260C_G263A_A264T_ANTISENSE | AAGACCATTCCGGCACATTGGTGAATTCGTCGCACATTGGG |
|  | T260C_G263A_A264T_SENSE | GGAAACCCAATGTGCGACGAATTCACCAATGTGCCGGAAT |
| H3 | T608C_A609G_G611A ANTISENSE | GTTGGGTTCTTGTAGAGATTCGTCTGCTCCTCTGCACTGTTG |
|  | T608C_A609G_G611A_SENSE | TTCCAACAGTGCAGAGGAGCAGACGAATCTCTACAAGAAC |
|  | G593A_ANTISENSE | GCTCCTCTGCATTGTTGGAATGATGGATTCCCC |
|  | G593A_SENSE | GGGGAATCCATCATTCCAACAATGCAGAGGAGC |

Table S3. Amino acid sequences for 3D-modelling of the monoclonal antibodies.

| **mAbs** | **Amino acid sequences** |
| --- | --- |
| **#23.3** | MGWSYIILFLVATATDVHSQVQLQQPGADLVKPGASVQLSCKASGYTFTSYWMHWVKQRPGQGLEWIGEINPSYGHTNYSEKFKSKATLTVDKSSSTAYMQLSSLTSEDSAVYYCARWDDDYYSWFAYWGQGTLVTVSAAKTTAPSVYPLAPVCGDTTGSSVTGGGGSGGGGSGGGGSTSMSRGQIVLTQSPAIMSASLGERVTMTCTASSSVSSSYLHWYQQKPGSSPKLWIYSTSSLASGVPARFSGSGSGTSYSLTISSMEAEDAATYYCHQYHRSPPTFGGGTKLEIK |
| **#11.4** | MEWSGVFIFLLSVTADVHSQVQLQQSGAELVRPGTSVKISYKASGSTFTNYWLGWVKQRPGHGLEWIGDIYPGGGYANYNEKFKGKATLTADTSSSTAYMQLSSLTSEDSAVYFCARHRYEYFDVWGAGTTVTVSSAKTTPPGGGGSGGGGSGGGGSTSMDMRAPAQIFGFLLLLFPGTRCDIQMTQSPSSLSASLGERVSLTCRASQDIGSSLNWLQQEPDGTIKRLIYATSSLDSGVPKRFSGSRSGSDYSLTISSLESEDFVDYYCLQYASSPYTFGGGTKLEIKRADAA |

The heavy chain (HC) and light chain (LC) of #23.3 are shown in bright orange and green, respectively; the HC and LC of #11.4 are shown in bright pink and blue, respectively. Complementarity-determining regions (CDRs) are highlighted in deep colours in both the HC and LC of the mAbs.


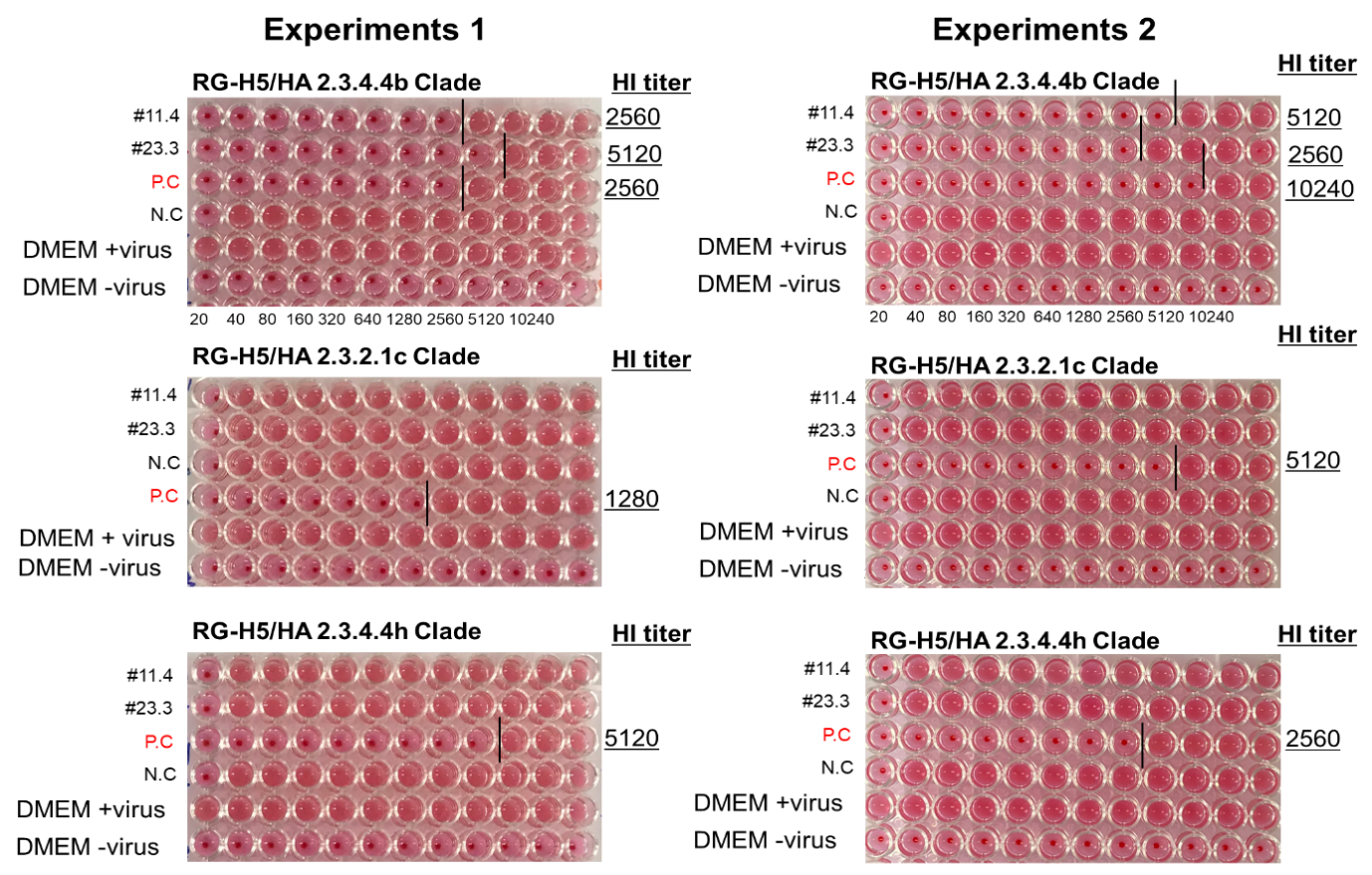


Figure S1. Haemagglutination results of microneutralisation assay. P.C., Chicken antiserum corresponded to the homologous virus; N.C., normal IgG; DMEM, Dulbecco's Modified Eagle Medium.


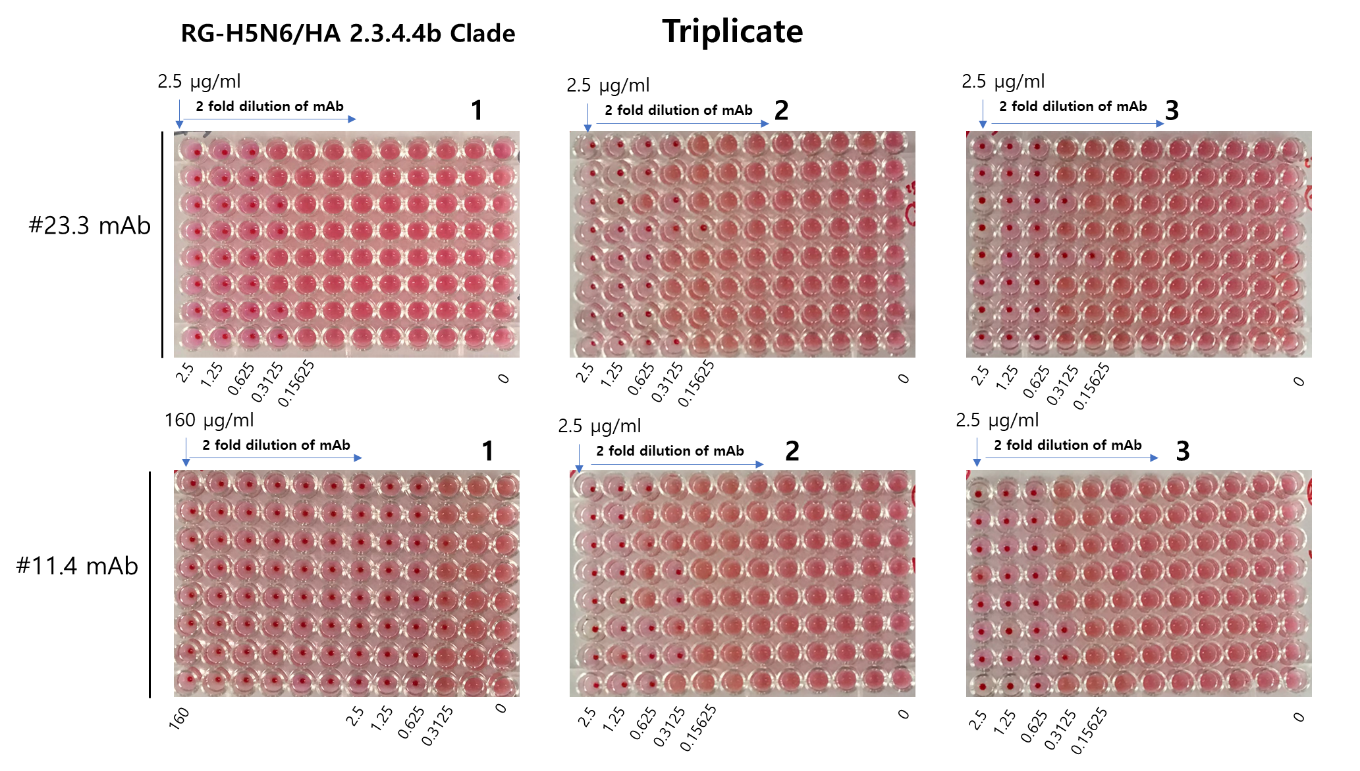


Figure S2. Haemagglutination results of inhibitory concentration 50% (IC_50_) values for #23.3 and #11.4 monoclonal antibodies (mAbs) against the RG-H5-HPAI-A/Anas/KR/2017/2.3.4.4b strain. The experiments were performed in triplicate.


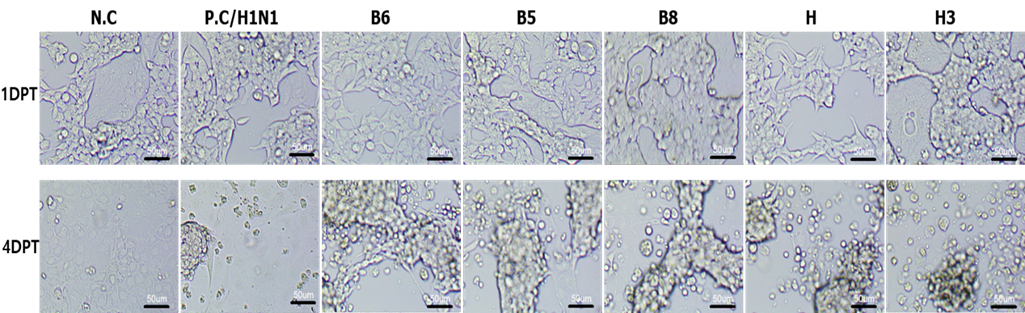


Figure S3. Rescue of the HPAIV H5 virus clades that contain haemagglutinin (HA) mutation sites. Scale bar, 50 µm. Original magnification, 100×; N.C., no plasmid transfection; P.C., H1N1 PR8 plasmids; DPT, days post-transfection.
